# The Longitudinal Relationship Between Cortisol Responses to Mental Stress and Leukocyte Telomere Attrition

**DOI:** 10.1210/jc.2016-3035

**Published:** 2016-12-14

**Authors:** Andrew Steptoe, Mark Hamer, Jue Lin, Elizabeth H. Blackburn, Jorge D. Erusalimsky

**Affiliations:** 1 Department of Epidemiology and Public Health, University College London, London WC1E 6BT, United Kingdom;; 2 School of Sport, Exercise, and Health Sciences, Loughborough University, Loughborough LE11 3TU, United Kingdom;; 3 Department of Biochemistry and Biophysics, University of California, San Francisco, California 94158; and; 4 Cardiff School of Health Sciences, Cardiff Metropolitan University, Cardiff CF5 2YB, Wales, United Kingdom

## Abstract

**Context:**

Chronic psychological stress has been associated with shorter telomeres, but the underlying mechanisms are poorly understood. One possibility is that the neuroendocrine responses to stress exposure are involved.

**Objective:**

To test the hypothesis that greater cortisol responsivity to acute stressors predicts more rapid telomere attrition.

**Design:**

We measured salivary cortisol responses to 2 challenging behavioral tasks. Leukocyte telomere length was measured at the time of mental stress testing and 3 years later.

**Participants:**

We studied 411 initially healthy men and women aged 54 to 76 years.

**Main outcome measure:**

Leukocyte telomere length.

**Results:**

Cortisol responses to this protocol were small; we divided participants into cortisol responders (n = 156) and nonresponders (n = 255) using a criterion (≥20% increase in cortisol concentration) previously shown to predict increases in cardiovascular disease risk. There was no significant association between cortisol responsivity and baseline telomere length, although cortisol responders tended to have somewhat shorter telomeres (*β* = −0.061; standard error, 0.049). But cortisol responders had shorter telomeres and more rapid telomere attrition than nonresponders on follow-up, after controlling statistically for age, sex, socioeconomic status, smoking, time of day of stress , and baseline telomere length (*β* = −0.10; standard error, 0.046; *P* = 0.029). The association was maintained after additional control for cardiovascular risk factors (*β* = −0.11; *P* = 0.031). The difference between cortisol responders and nonresponders was equivalent to approximately 2 years in aging.

**Conclusions:**

These findings suggest that cortisol responsivity may mediate, in part, the relationship between psychological stress and cellular aging.

## Teaser

We studied cortisol responses to acute mental stress and found that larger responses predicted accelerated leukocyte telomere attrition over 3 years in healthy older men and women.

Telomeres are complexes of DNA and proteins situated at the ends of chromosomes and that protect the genomic DNA of eukaryotic cells (1). Telomeres shorten with each cell division, and telomere length is a marker of cellular aging. Telomere function is impaired when shortening becomes critical, leading to cell senescence, genome instability, and apoptosis. Leukocyte telomere length is associated with increased risk of cardiovascular disease, cancers, diabetes, dementia, and all-cause mortality (2–4). These relationships have been confirmed by studies of inherited telomere syndromes (5) and by Mendelian randomization studies (6).

Several environmental and lifestyle factors are associated with telomere shortening, including smoking, obesity, and physical inactivity (7). There is growing interest in the relationship of leukocyte telomere length with psychiatric conditions and psychological stress, as well. Large-scale investigations indicate that individuals with major depressive disorder have shorter telomeres independently of demographic factors and health behaviors, although findings across studies have been variable (8). Anxiety disorders may also be associated with reduced telomere length (8), and a meta-analysis of 22 studies documented a small, statistically significant relationship between greater perceived stress and shorter telomeres (9). Exposure to early life adversity has been linked with reduced telomere length in some studies (10) but not in all (11). Associations with low social support (12) and hostility (13) have also been described.

Evaluation of the importance of links between stress exposure, mental health, and telomere dynamics would be strengthened by better understanding of potential underlying mechanisms. Unhealthy habits such as smoking, excessive alcohol consumption, and inactivity might play a role, but many studies have observed associations with leukocyte telomere length after these factors have been taken into account (8, 9, 14). The physiological responses associated with mental stressors may also be involved. Cortisol plays a central role in the stress response because of its multiple effects on immune, metabolic, and vascular processes. Animal studies indicate that embryonic exposure to corticosteroids elicits increased oxidative stress and shorter telomeres in later life (15). There are large individual differences in the magnitude of cortisol responses to standardized mental stress tests, and these reflect variations in the capacity of neuroendocrine regulatory processes to adapt to challenge. A few studies have shown that larger cortisol responses to mental stress are associated with shorter telomeres in adults and children (16–18). For example, Tomiyama *et al.* (19) administered a standardized mental stress protocol to 28 caregivers for people with Alzheimer’s disease and control subjects, and found that telomeres were shorter in individuals who manifested greater cortisol stress responses. However, these studies of telomeres and stress physiology have been cross-sectional. It is possible that heightened cortisol responsivity drives telomere attrition or, conversely, that greater cortisol responses are characteristic of people with shorter telomeres. Null associations have also been described (20).

In this study, we evaluated the relationship between cortisol responses to mental stress and differences in telomere length measured at the time of mental stress testing and 3 years later. We tested the hypothesis that cortisol stress responders would show greater telomere attrition over time than nonresponders. This hypothesis was examined in a sample of healthy men and women aged 54 to 76 years, because biological aging processes are particularly relevant to disease risk as people progress into older age. We used a measure of cortisol responses to mental stress tests that has been shown to predict the progression of subclinical coronary atherosclerosis as indexed by coronary calcification (21), and the development of hypertension (22). Our analyses also took into account sociodemographic and physiological factors that might also contribute to telomere shortening over time.

## Materials and Methods

### Participants

We analyzed data from the Heart Scan Study, a sample of 543 men and women of white European origin of the Whitehall II epidemiological cohort recruited between 2006 and 2008 to investigate physiological responsivity to mental stress testing and subclinical coronary artery disease (21). Participants were selected as having no history of coronary heart disease and no previous diagnoses or treatment of hypertension, diabetes, inflammatory diseases, or allergies. We used civil service employment grade as an indicator of socioeconomic status (SES), and recruitment was stratified to include men and women from higher, intermediate, and lower employment grades. The women in the study were postmenopausal. Participants were invited for reassessment 3 years after mental stress testing (mean interval, 1087 days). Ethical approval was obtained from the University College London Hospital Committee on the Ethics of Human Research, and all participants gave signed informed consent. All procedures were carried out in accordance with approved guidelines.

**Figure 1.**
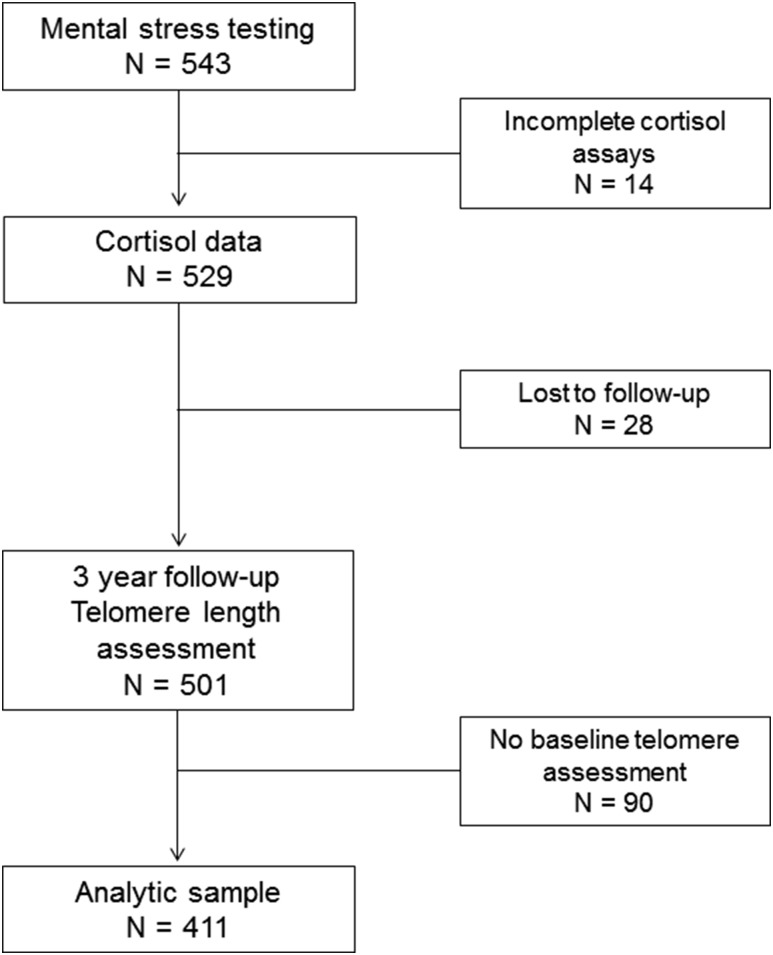
Flowchart of study participation.

Figure 1 shows a flowchart summarizing participant progression through the study. Telomere length was measured in 501 respondents (92.3%) an average 36.2 months after stress testing. Of these, 411 also had telomere length measures at the time of stress testing because assessments were not introduced at the start of data collection. They constituted the sample for this study. There were no differences on any measures between individuals included and not included in the telomere length analyses.

### Laboratory mental stress testing

We tested participants individually in a light- and temperature-controlled laboratory, with sessions beginning either in the morning between 8:30 and 9:30, or in the early afternoon between 13:30 and 14:30. Participants were instructed not to drink caffeinated beverages or smoke for at least 2 hours before testing, to avoid vigorous exercise and alcohol from the previous evening, and not to have taken any anti-inflammatory or antihistamine medication for the 7 days before testing. They were rescheduled if they reported colds or other infections on the day of testing. At the start of the session, participants’ height, weight, and waist and hip circumferences were measured using standardized techniques, and body mass index (BMI) was computed. After a 30-minute rest period, baseline blood pressure (BP) was measured with an automated Lifesource UA-779 digital monitor (A&D Medical, San Jose, CA), a blood sample was drawn, and a saliva sample was taken using salivettes (Sarstedt, Leicester, United Kingdom). Two behavioral tasks designed to induce mental stress were then administered in random order (21, 23). Both tasks were performed for 5 minutes. One was a computerized version of the Stroop color-word interference task, which involved successive presentation of target color words (*e.g.*, red, blue) printed in another color. Four names of colors were printed in incongruous colors at the bottom of the computer screen and participants were requested to press the computer key that corresponded to the position at the bottom of the screen of the name of the color in which the target word was printed. The rate of presentation of stimuli was adjusted to the performance of the participant to ensure sustained demands. The second task was mirror tracing, which involved tracing with a metal stylus a star that could only be seen in mirror image. Each time the stylus came off the star, a mistake was registered and a loud beep was emitted by the apparatus (Lafayette Instruments Corp., Lafayette, IN). Participants were told that the average person could complete 5 circuits of the star in the available time. These tasks were selected because they have been shown to stimulate similar appraisals of involvement and engagement from participants across the social gradient. A second saliva sample was taken immediately after tasks, with further samples collected at 20, 45, and 75 minutes after tasks.

### Biological measures

Saliva samples were analyzed for cortisol concentration using a time-resolved immunoassay with fluorescence detection at the Technical University Dresden, as described previously (24, 25). The intra- and interassay coefficients of variation were <8%. Total and high-density lipoprotein (HDL) cholesterol levels in serum stored at 4°C were measured within 72 hours of collection, using enzymatic colorimetric methods. Glycated hemoglobin was measured using a Tosoh G7 high-performance liquid chromatography analyzer (San Francisco, CA) calibrated to Diabetes Control and Complications Trial standards. An adaptation of the method first described by Cawthon (26) was used for the assessment of leukocyte telomere length. Genomic DNA was extracted from peripheral blood mononuclear cells in a QIAcube workstation (baseline) or manually (follow-up) with the QIAamp DNA blood mini kit (Qiagen, Crawley, United Kingdom), according to instructions of the manufacturer, and stored in 10 mmol/L Tris-hydrochloride, 0.5 mmol/L ethylenediamine tetraacetate, at pH 9.0 at −20°C (baseline) or −80°C (follow-up). The relative mean telomere length was measured by a monochrome multiplex quantitative real-time polymerase chain reaction (PCR) assay with a Bio-Rad CFX96 Real-Time PCR Detection System (Hemel Hempstead, United Kingdom) for samples obtained at the time of mental stress testing, and with a Roche Lightcycler 480 real-time PCR machine (Roche Diagnostics, Indianapolis, IN) on follow-up (27). Reactions containing serial dilutions of a reference DNA standard were included in each PCR plate to generate the telomere and *β*-globin gene standard curves required for quantitation, and relative mean telomere length, expressed as a telomere–to–*β*-globin gene ratio, was derived. The coefficient of variation of these assays was 2.3%.

### Data reduction and statistical analysis

The mental stress protocol in this study did not generate large cortisol responses, with many respondents not showing an increase after tasks. Cortisol stress responsivity, therefore, was quantified by calculating differences scores between the baseline cortisol concentration and the samples obtained both immediately after tasks and 20 minutes later. Individuals who showed a ≥1 nmol/L increase (equivalent to a 20% increase) between baseline and either sample were defined as cortisol responders, and the remainder as nonresponders. Differences between the responder groups at baseline were analyzed using analysis of variance and *χ*^2^ methods for continuous and categorical variables, respectively. The cortisol profiles across the mental stress testing session of responder and nonresponder groups were compared using repeated measures analysis of variance with sample as the within-person factor and responder status as the between-person factor. Associations between cortisol stress responsivity and telomere length at baseline were analyzed using multivariable regression, including age, sex, grade of employment, smoking status, and time of stress testing (morning or afternoon) as covariates. A similar method was used to analyze associations between cortisol stress responsivity and follow-up telomere length, except, in this case, baseline telomere length was included as a covariate. Results are presented as standardized regression coefficients (*β*) with standard errors.

In a sensitivity analysis, we added cardiovascular risk factors (systolic BP, BMI, total and HDL cholesterol, and glycated hemoglobin) to the model; these factors were not included in the main model because missing data on some variables reduced the sample size.

Absolute measures of telomere length can vary across laboratories, but rankings of relative length are highly correlated (28). In view of the different systems used at baseline and follow-up, we therefore computed standardized telomere length scores for the 2 time points. However, repeating the analyses with standardized, as opposed to absolute, values generated identical statistical findings, so the latter are presented in the Results.

## Results

The 411 participants included 156 cortisol responders and 255 nonresponders. The characteristics of these 2 groups are summarized in Table 1. Participants generally had favorable risk profiles, with few smokers, BP and glycated hemoglobin in the healthy range, and no marked elevation of BMI or cholesterol. There were no differences in any sociodemographic or physiological factors between the 2 groups. There was a nonsignificant tendency of cortisol responders to be more likely to have undertaken mental stress testing in the afternoon compared with nonresponders (*P* = 0.096), so time of day was included as a covariate in the analyses.

**Table 1.** **Characteristics of Cortisol Responders and Nonresponders**

Variable	Nonresponders (n = 255)	Responders (n = 156)	*P* Value
Age, y	63.1 ± 5.6	63.6 ± 5.7	0.36
Men, %	47.5	48.1	0.52
Grade of employment, %			
Higher	39.6	30.8	0.36
Intermediate	34.5	44.9	
Lower	25.9	24.4	
Current smoker, %	6.3	5.8	0.51
Baseline systolic BP, mm Hg	124.8 ± 14.5	126.8 ± 15.4	0.18
Body mass index, kg/m^2^	25.7 ± 4.3	26.1 ± 3.7	0.26
Total cholesterol, mmol/L	5.33 ± 0.95	5.34 ± 0.91	0.89
HDL cholesterol, mmol/L	1.70 ± 0.47	1.66 ± 0.47	0.72
Glycated hemoglobin, %	5.48 ± 0.39	5.46 ± 0.40	0.76
mmol/mol	36.3	36.2	
Stress testing in afternoon, %	57.3	66.0	0.096
Follow-up interval, d	1073 ± 62.6	1068 ± 73.3	0.48

Data given as mean ± standard deviation unless otherwise indicated.

**Figure 2.**
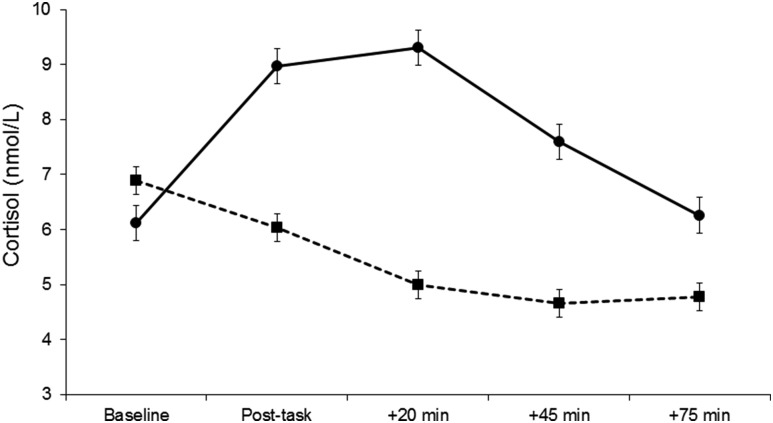
Mean salivary cortisol concentration at baseline, immediately after behavioral tasks (post-task), and 20, 45, and 75 minutes after tasks in cortisol responders (solid line) and cortisol nonresponders (dashed line). Error bars are standard errors of the mean.

Cortisol concentrations in the responders and nonresponders to behavioral challenge are shown in Fig. 2. There was a robust interaction between responder group and trial (*P* < 0.001). Cortisol concentrations were similar in the 2 groups at baseline. But whereas the responder group showed an average 47% increase in salivary cortisol after tasks, values declined steadily in the nonresponder group. Even 75 minutes after mental stress tests had been completed, cortisol concentration remained more than 30% higher in the responder group than nonresponder group.

The mean telomere–to–*β*-globin gene ratio averaged 0.992 ± 0.07 at baseline, and 0.894 ± 0.15 at follow-up. This indicates a significant decrease in telomere length over the 3-year interval (*P* < 0.001). Telomere lengths at the 2 time points were moderately correlated (*r* = 0.31; *P* < 0.001). There was a small positive association between baseline telomere length and change over time (*r* = 0.20), indicating that participants with longer telomeres showed greater shortening. Telomere length on follow-up was inversely associated with age (*P* < 0.001), and was shorter in men than women (*P* < 0.001).

**Figure 3.**
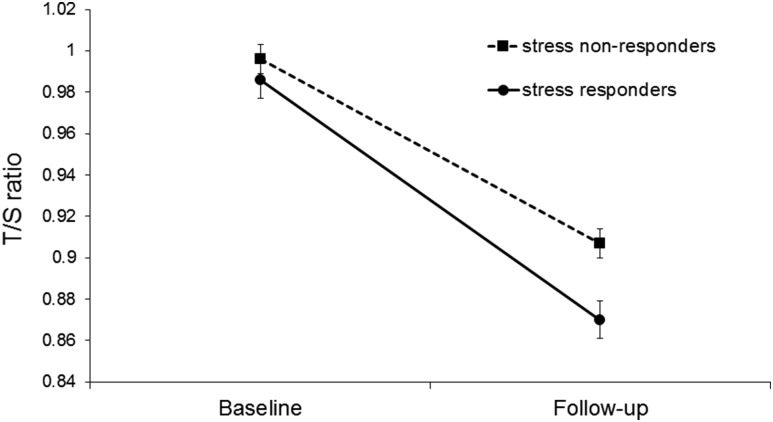
Mean telomere length (T/S ratio) in cortisol stress responders (solid line) and nonresponders (dashed line) at baseline and 3-year follow-up. Values are adjusted for age, sex, grade of employment, smoking status, and baseline telomere length. Error bars are standard error of the mean. Telomere length was significantly different between cortisol responder and nonresponder groups at follow-up (*P* = 0.016). T/S, telomere–to–*β*-globin gene.

The relationship between cortisol stress responsivity and telomere length at baseline was negative, though not significant [*β* = −0.061; standard error (SE), 0.049; *P* = 0.22]. But we found that cortisol stress responsivity was associated with shorter telomere length on follow-up after adjustment for baseline telomere length, age, sex, grade of employment, smoking status, and time of stress testing (*β* = −0.10; SE, 0.046; *P* = 0.029; Table 2). The other independent predictors of shorter telomeres on follow-up were older age, male sex, and shorter telomere length at baseline. Figure 3 illustrates the pattern of change in telomere length over time in cortisol responders and nonresponders to stressors, showing the greater shortening over time in stress responders. There was no interaction between time of stress testing and cortisol responsivity in predicting telomere length on follow-up.

**Table 2.** **Predictors of Follow-Up Leukocyte Telomere Length**

Predictor	*B*	*β* (SE)	*P* Value
Cortisol stress responsivity	−0.031	−0.10 (0.046)	0.029
Age	−0.005	−0.19 (0.047)	<0.001
Sex	0.055	0.18 (0.046)	<0.001
Grade of employment	0.009	0.05 (0.046)	0.32
Smoking status	0.013	0.02 (0.046)	0.66
Time of stress testing	−0.004	−0.01 (0.047)	0.77
Baseline telomere length	0.560	0.28 (0.046)	<0.001

The association was unchanged in the sensitivity analysis, which included baseline systolic BP, BMI, total and HDL cholesterol, glycated hemoglobin, and time interval between baseline and follow-up (n = 378; *β* = −0.11; SE, 0.049; *P* = 0.031).

## Discussion

In this study, we tested the notion that cortisol responses to mental stress would be associated with the rate of telomere attrition. We found that healthy, men and women in late middle age who responded to standardized behavioral challenges with larger increases in salivary free cortisol showed greater shortening of leukocyte telomeres over 3 years. This association was independent of baseline telomere length, age, sex, SES defined by grade of employment, smoking, cardiovascular risk factors (BP, cholesterol, BMI, glycated hemoglobin), and length of follow-up. The difference in telomere attrition between cortisol responders and nonresponders corresponded to 107 base pairs on follow-up, indicating a difference of approximately 2 years in aging (29).

The cortisol responses during mental stress testing in this study were small. A major purpose of the study from which these data were drawn was to evaluate SES differences in stress reactivity and recovery (23). Consequently, the task protocol was designed to be perceived as equally stressful across the SES spectrum and was selected after pretesting on this criterion. It did not involve socially evaluative tasks such as the Trier Social Stress Test that are known to elicit large cortisol responses (30), because such tasks are often appraised differently by higher and lower social status individuals, compromising any differences in physiological responsivity. The range of individual differences, as well as absolute magnitude of cortisol responses, therefore, was smaller than in some other investigations. However, the value of the cortisol responder categorization adopted here has been endorsed by evidence that individuals classified as cortisol responders show an increased risk of incident hypertension (22) as well as more rapid progression of subclinical coronary artery disease as indexed by coronary artery calcification (21). Brief cortisol responses to short-term tasks are of little significance in themselves. However, the magnitude of acute cortisol responses is positively associated with cortisol output in everyday life (31). If these responses are representative of people’s habitual profile of cortisol when confronted by the challenges of everyday life, they may contribute to chronic neuroendocrine activation that could have deleterious health consequences.

Research relating telomere length with measures of cortisol output at rest have produced mixed results (32, 33), suggesting that relating individual differences in cortisol responses to standardized mental stress with telomere length may be a valuable strategy. Epel *et al.* (16) found that urinary cortisol concentration collected over a night following a behavioral stress battery was inversely associated with telomere length in healthy women. A study of older female caregivers of partners with dementia showed relationships between telomere length and cortisol responses to behavioral challenge (19), whereas work with children as young as 5 to 6 years has demonstrated that cortisol reactivity to mildly stressful tasks is inversely correlated with telomere length (17, 18). By contrast, a study of older men and women in Finland showed no associations between telomere length and cortisol responses to acute stress exposure, but this is difficult to interpret because stress testing took place an average of 2.1 years after telomere assays (20). Our study builds on these findings by establishing a longitudinal relationship, because cortisol responsivity predicted telomere shortening over time. The results are also consistent with longitudinal clinical studies indicating that telomere length is shorter during active Cushing’s syndrome than when patients are in remission (34).

A puzzling feature of our results is that no association was present between cortisol responsivity and telomere length at baseline. There was a negative association between cortisol responsivity and baseline telomere length, but it was not statistically significant. It is potentially relevant that the studies of adults that have shown associations between cortisol responsivity and telomere length have focused on individuals exposed to chronic stressors such as caregiving or having children with severe disabilities (16, 19). To our knowledge, no association has previously been observed in general population samples of the type involved in this study (20). It is possible that in our sample of relatively healthy older men and women, these associations only emerged after several years.

We found a positive correlation between baseline telomere length and the magnitude of the change in length over time. Regression to the mean has been put forward as the explanation of this phenomenon (35). However, regression to the mean is unlikely to be the explanation for the association with cortisol stress responsivity, because, if anything, cortisol responders had slightly shorter telomeres at baseline. Regression to the mean, therefore, would operate against the effects observed here.

The mechanisms underlying these associations have yet to be defined in detail. Telomere length is regulated dynamically and does not decrease monotonically with advancing age (1). Faster telomere attrition over time may result from several causes, including the expansion of leukocyte subsets that occurs during inflammation and immunological responses, a decrease in telomerase activity, and oxidative stress (27). Although cortisol responses might be expected to inhibit inflammation, simultaneous heightened inflammation and cortisol is common in response to behavioral stress. A reason for this might be because glucocorticoids have proinflammatory effects under some circumstances. *In vitro* administration of glucocorticoids induces cytokine overexpression and NF-*κ*B activation in isolated macrophages (36), whereas pretreatment with cortisol has been found to enhance interleukin-6 responses to endotoxin (37). Cortisol administration *in vitro* also appears to reduce telomerase activity (38). Frank *et al.* (39) have proposed that glucocorticoid responses to stress may be neuroendocrine warning signals to the innate immune system, sensitizing neuroinflammatory processes even after the corticosteroid response has dissipated. The combined effect of reduced telomerase activity and oxidative stress would impinge negatively on the maintenance of telomere length, particularly in the context of chronic inflammation, thus providing a plausible explanation for our findings.

This study has several limitations. The participants were middle-aged and older white European men and women with no serious chronic illness, and results may not generalize to other groups. Telomere length was measured in peripheral blood mononuclear cells, and values may differ in lymphocyte subpopulations. Measures were also made with 2 different PCR machines at the 2 time points; although this might affect comparisons of absolute values on the 2 occasions, it does not affect the relative changes that are central to these results, so findings were the same with standardized measures of telomere length. The cortisol responses were less substantial than those recorded with socially evaluative stress testing, reducing the variability in responsivity profiles. We did not include a no-stress control group in this study, because we have previously found that the measurement protocol itself does not induce physiological responses (40).

A strength of the study is that our findings were obtained in a well-characterized longitudinal population cohort, with a rather larger sample than has previously been evaluated for cortisol responses to acute mental stress and telomere length. The results may have implications for understanding the pathways through which social and environmental factors and mental ill health impact cellular aging. If associations between stress exposure and mental distress and telomere length are mediated through cortisol responsivity, it is possible that the effects of mental stress on cellular aging might be reduced not only by modifying stress exposure (which is not necessarily practical) but also by attenuating the physiological components of the stress response.

In conclusion, the results of this study strongly suggest that heightened cortisol responsivity to psychological stress is associated with accelerated cellular aging as indexed by leukocyte telomere length. This indicates that heightened cortisol responsivity is not simply a consequence of more advanced cellular aging but may contribute to the cellular aging process.
